# Supplementary material for: Functional evolution of Lepidoptera olfactory receptors revealed by deorphanization of a moth repertoire
Source: Nat Commun. 2017 Jun 5;8:15709. doi: 10.1038/ncomms15709 (PMC5465368; doi:10.1038/ncomms15709)
Supplement: Supplementary Information — Supplementary Figures, Supplementary Tables and Supplementary References [file ncomms15709-s1.pdf]

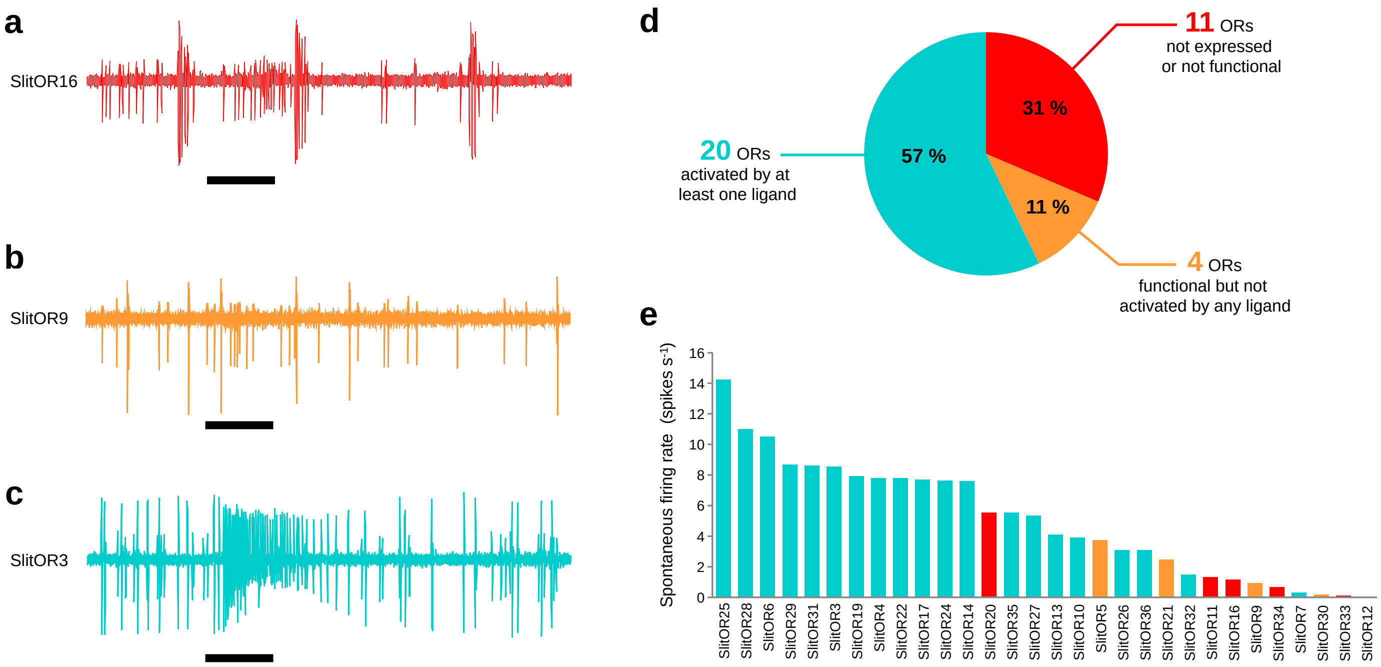

Supplementary Figure 1. Expression of SlitORs in the empty neuron system

(**a**) Example of single-sensillum recording from an ab3 sensilla of a transformed fly expressing a non-functional receptor (SlitOR16). Action potentials from the ab3A OSN (which expresses the SlitOR) are those with the largest amplitude. The spontaneous activity, with bursts of 5-6 consecutive spikes, is characteristic of an ab3A OSN with no OR expressed. The black bar represents the duration of the stimulation (500 ms). (**b**) Example of a functional receptor not activated by any ligand (SlitOR9). The spontaneous activity is regular, but there is no increase in the frequency of action potentials during the stimulation. (**c**) Example of a deorphanized receptor (SlitOR3). The frequency of action potentials increases during stimulation by the ligand of the receptor. (**d**) Pie chart showing the ‘success rate’ of SlitOR expression in the empty neuron system. (**e**) Mean spontaneous firing rates elicited by SlitORs when expressed in the empty neuron system. Cyan, orange and red colours refer to the three categories indicated in the pie chart.

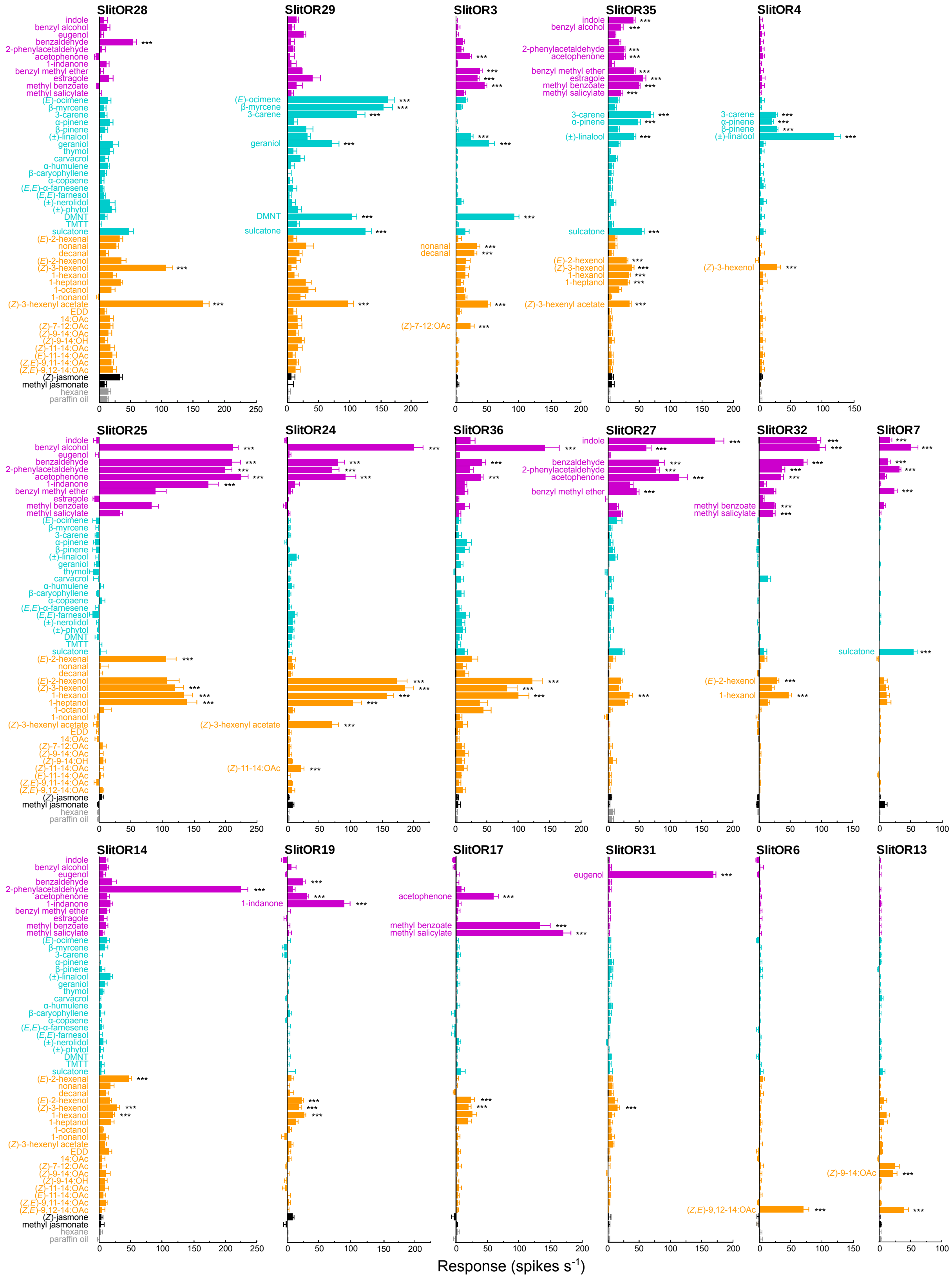

Supplementary Figure 2. Response spectra of SlitORs expressed in the empty neuron system

Odorants were presented at a 10^{-2} dilution (except pheromones, diluted at 10^{-3}). Odorants are classified depending on their chemical class (magenta, aromatics; cyan, terpenes; orange, aliphatics; black, unclassified). Solvents are in grey. Error bars indicate s.e.m. ***, significantly different from the response to solvent (Kruskal-Wallis ANOVA followed by a Dunn's post hoc test, $P < 0.001$, n = 5-10).

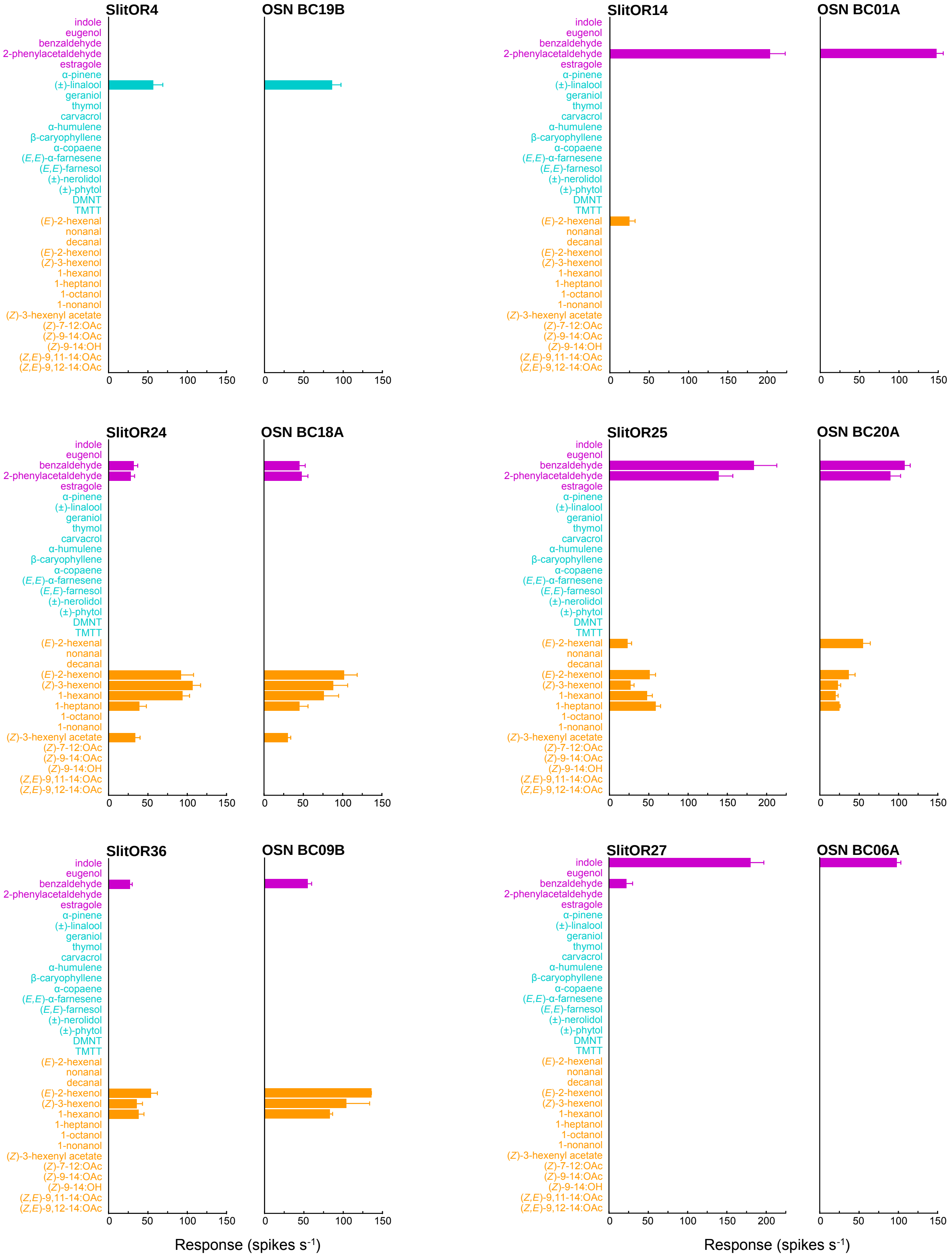

Supplementary Figure 3. Comparison of response spectra of SlitORs and *S. littoralis* OSNs

Response spectra of SlitORs expressed in the empty neuron system (10^{-3} odorant dilutions) are compared with response spectra of *S. littoralis* female OSNs[1]. Only the responses to odorants tested in both studies are presented. Odorants are classified as in Fig. 2. Error bars indicate s.e.m.

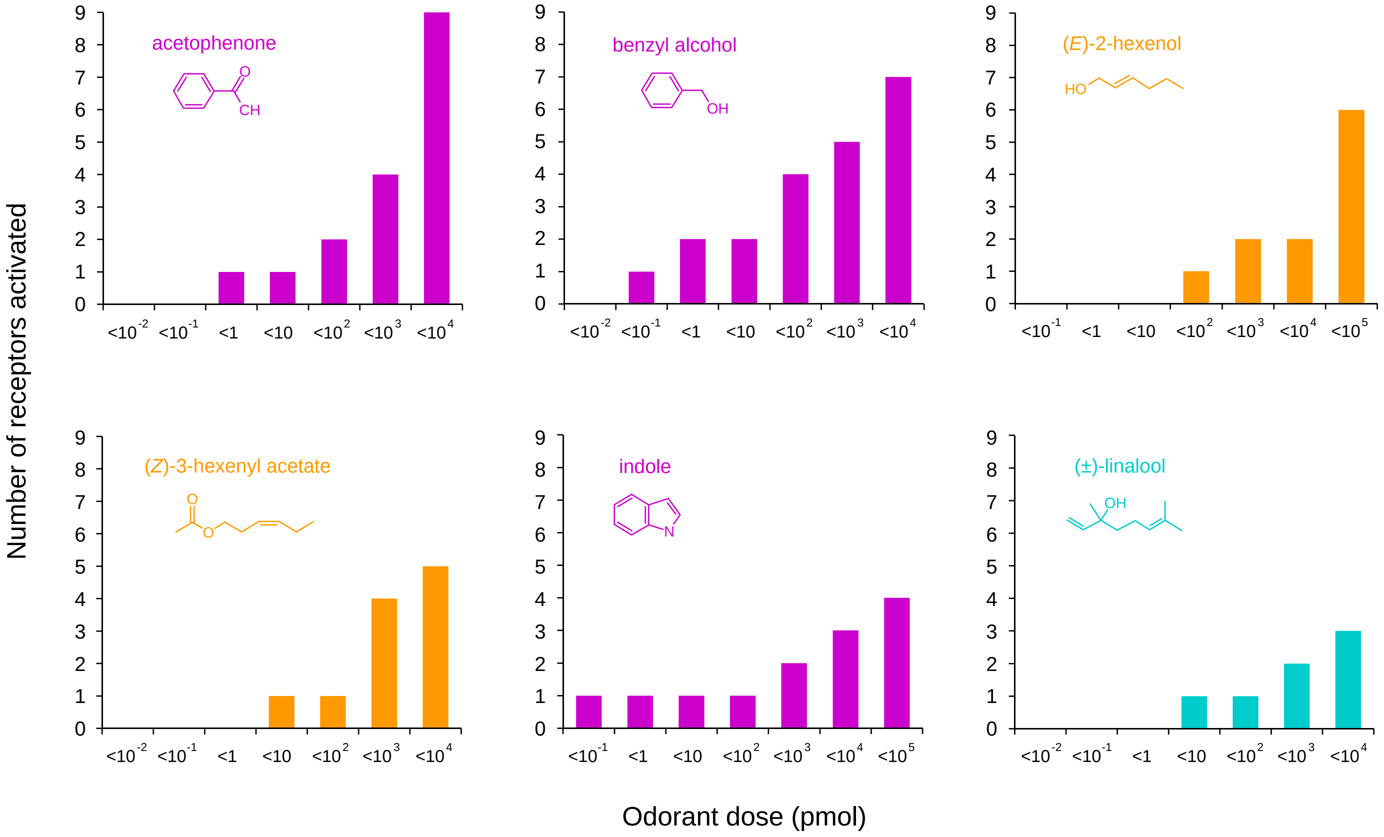

Supplementary Figure 4. Coding of odorant quantity

Histograms presenting the number of SlitORs significantly activated by six odorants from the panel along a range of stimulus quantities.

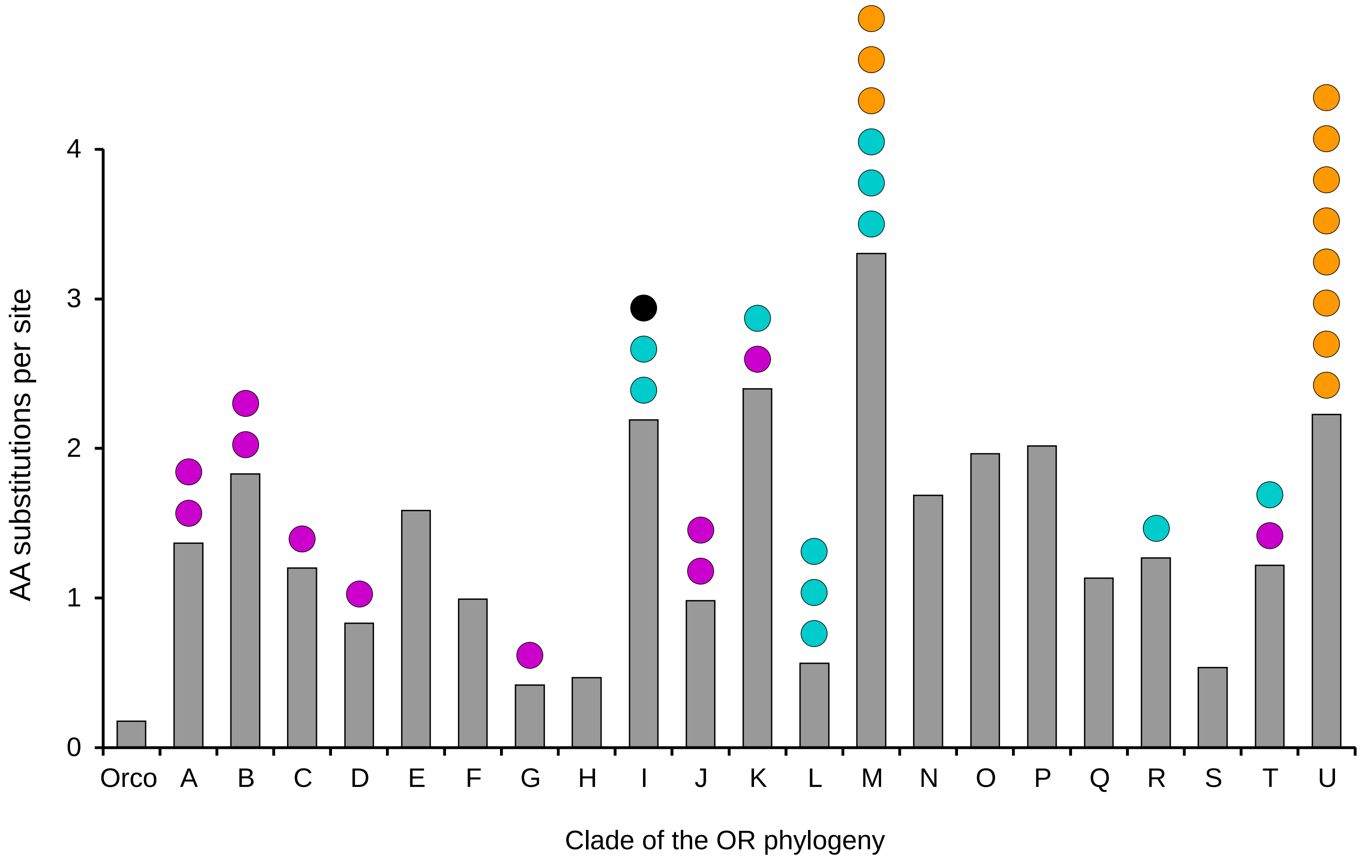

Supplementary Figure 5. Evolutionary rates in the different lepidopteran OR lineages

Histogram of the mean phylogenetic distance (number of expected amino acid substitutions per site estimated by maximum-likelihood) in each clade of the phylogeny of lepidopteran ORs (Fig. 5). Circles represent the number of ORs deorphanized in each clade, and their colours correspond to the chemical class of the best ligand identified (magenta, aromatics; cyan, terpenes; orange, aliphatics; black, unclassified).

Supplementary Table 1. Methods used for the generation of *UAS-SlitOR* constructs and fly lines

Receptor	Forward primer	Reverse primer	Destination vector	Transgenesis method	Functionality in the empty neuron
**SlitOR1**	ATGGACTCGAATGTAGATAAGA	TTACTTTTCATTGACATTAACTAGAAGAG	pUASgw-HA.attB	PhiC31	not expressed
**SlitOR3**	GGGATCATTTACCGTTCCAA	CAATTCCTAGATGGCTACTAAAGAACA	pUAST.attB	PhiC31	functional (deorphanized)
**SlitOR4**	CGTTGAGAACGGTCAAAGGTTTGTC	TGGCCGCTAATTGTAGGCTTTGTG	pUAST.attB	PhiC31	functional (deorphanized)
**SlitOR5**	CCAGACGAAGCAGACACCAATTCTA	CACTCATACGTTCATACACTGCAAG	pUAST	P-element	functional (not deorphanized)
**SlitOR6**	ATGGGTTTAAAAAAGTTTCTTTTTG	CTCAAATGCTGCGTAGGAAGGTG	pUAST	P-element	functional (deorphanized)
**SlitOR7**	ATGCCCAAACCACTGTTATTTG	TTATCTTCCGTACACTGTCTGC	pUASgw-HA.attB	PhiC31	functional (deorphanized)
**SlitOR8**	ATGGAATCCCATCAAATAGA	TTACTCTTTCTGAAAGTGCTGG	pUASgw-HA.attB	PhiC31	not expressed
**SlitOR9**	*synthesized in vitro*		pUAST.attB	PhiC31	functional (not deorphanized)
**SlitOR10**	ATGGAGGCAGAAAAAAACACG	TTATTCTGTGTTCACCCTGTTCA	pUASgw-HA.attB	PhiC31	functional (not deorphanized)
**SlitOR11**	AAATGTTTAACTTAGATACGTATG	TTTAAAATGTGCGCAAGAAAGCGA	pUAST	P-element	expressed but not functional
**SlitOR12**	ATGGAAGAAGAACCTCTGTTAA	TTATGGAGGCGAACCATACAA	pUASgw-HA.attB	PhiC31	expressed but not functional
**SlitOR13**	ATGGATATAAAATTGTCATCAGT	CTTATTCTTCTTCATCGGCAAC	pUAST	P-element	functional (deorphanized)
**SlitOR14**	ATGACTGAAACACGTCCT	TTACTGCTGTTTTTGACT	pUASgw-HA.attB	PhiC31	functional (deorphanized)
**SlitOR15**	AACCATGCCGTCGGATCAAT	ACGTAGGAAGGCCGAAAACA	pUAST.attB	PhiC31	not expressed
**SlitOR16**	CGGTGGCATCAATTCTCCATCCCAA	CGTAGAAAGCAACCACAGGAAATGTTAGCA	pUAST	P-element	expressed but not functional
**SlitOR17**	*synthesized in vitro*		pUAST.attB	PhiC31	functional (deorphanized)
**SlitOR18**	ATGGAAATGAAATCAGATATTC	TCAAGCAGTAATCAAAGTGAAG	pUAST	P-element	not expressed
**SlitOR19**	ATGAAAAACCATTACATCTTGAA	TTACGAAGTTTGCGCATAAAAC	pUASgw-HA.attB	PhiC31	functional (deorphanized)
**SlitOR20**	CTGTCAGTCCTGCACATTCT	GTATTCAGGCACTTGGCATCTC	pUAST.attB	PhiC31	expressed but not functional

**SlitOR21**	ATGGACAACTTTTCTGGGTCAT	TTAATACAACACGGAAAATACT	pUASgw-HA.attB	PhiC31	functional (not deorphanized)
**SlitOR22**	TGGCAGTGAAAAGCCTAACTGT	ATTCGGTGCGTAGACGACAA	pUAST.attB	PhiC31	functional (not deorphanized)
**SlitOR23**	CTCTTCAGTACCAGCGCTATGTG	CCTGCTGGTTCATACTGAATAGTGT	pUAST.attB	PhiC31	not expressed
**SlitOR24**	ATGAGGGTCTTAAGCCATGTGT	TCATTCGTGGCTCATCGTTAGA	pUASgw-HA.attB	PhiC31	functional (deorphanized)
**SlitOR25**	ATGGGTCTCATCAAAAAC	TTAATCATGGCGGAAACG	pUASgw-HA.attB	PhiC31	functional (deorphanized)
**SlitOR26**	*synthesized in vitro*		pUAST.attB	PhiC31	functional (not deorphanized)
**SlitOR27**	AAATGATAATTCTTAATGAAAATATGAAAACC	CTGGATTATGTATTTTGTAAGCA	pUAST.attB	PhiC31	functional (deorphanized)
**SlitOR28**	TGCAACATGACGTCTCTTTATAG	CCAGCGTCCATGTTGTAGATTC	pUAST.attB	PhiC31	functional (deorphanized)
**SlitOR29**	ATGAATTCGTTTCTTCAGAGC	TTATTTCCTCAACAAAGTGTAG	pUASgw-HA.attB	PhiC31	functional (deorphanized)
**SlitOR30**	ATGGATGCAACATGTTTAAATT	TTATTTCCAAGCTGCGTTCA	pUASgw-HA.attB	PhiC31	functional (not deorphanized)
**SlitOR31**	TCAAAATGGAAGATAATGTAGCA	TCCCCGCTTCTTTCTATCT	pUAST.attB	PhiC31	functional (deorphanized)
**SlitOR32**	ATGGTCTCCTCAGAAGACCT	TTAGTGCGTTTGGTTCAAAACT	pUASgw-HA.attB	PhiC31	functional (deorphanized)
**SlitOR33**	ATGAGTAATCAAATAAAA	TCAATAGAATACTGACAC	pUASgw-HA.attB	PhiC31	expressed but not functional
**SlitOR34**	ATGAATTTCTTCAGAAACCCAGAA	TTAGTACAGCACAGAAAACAGAG	pUASgw-HA.attB	PhiC31	expressed but not functional
**SlitOR35**	CAAACCCCAAAAGATGTGGA	GCAACTCATAATCGGTTATTTC	pUAST.attB	PhiC31	functional (deorphanized)
**SlitOR36**	ATGTTGACCTTTCATGAAATC	TTACTTCATAGTAGACCTAAG	pUASgw-HA.attB	PhiC31	functional (deorphanized)

Supplementary Table 2. Synthetic compounds used for electrophysiology experiments

Compound	CAS	Provider	Purity	Detection by antennae	Emission source	Effect on behaviour
**indole**	120-72-9	Aldrich	99	2 ORN classes[1,2], active in GC-EAD[3]	Herbivore-Induced Volatile in cotton[4].	Present in an oviposition-deterrent blend[3]
**benzyl alcohol**	100-51-6	Aldrich	99	Active in GC-EAD[5]	Lilac flowers[5]	
**eugenol**	97-53-0	Aldrich	98	1 ORN class[2]	Larval frass[6]	Present in an oviposition-deterrent blend[6]<br>Repellent for caterpillars[7]
**benzaldehyde**	100-52-7	Aldrich	99.5	8 ORN classes[1], active in GC-EAD[5]	Larval frass[6], lilac flowers[5] , cotton[8]	Present in an oviposition-deterrent blend[6]
**2-phenyl acetaldehyde**	122-78-1	Aldrich	98	10 ORN classes[1], active in GC-EAD[5]	Lilac flowers[5]	
**acetophenone**	98-86-2	Acros	99	Active in EAG7 and in GC-EAD[5]	Larval frass[6], lilac flowers[5]	
**1-indanone**	83-33-0	Aldrich	99	1 ORN class[2]	Larval frass[6]	
**benzyl methyl ether**	538-86-3	Aldrich	98	Active in GC-EAD[5]	Lilac flowers[5]	
**estragole**	140-67-0	Sigma	96	2 ORN classes[1], active in GC-EAD[5]	Lilac flowers[5]	
**methyl benzoate**	93-58-3	Acros	97	Active in GC-EAD[3]	Damaged cotton plants[3]	
**methyl salicylate**	119-36-8	Sigma	99	2 ORN class[9]		
**(*E*)-ocimene**	3779-61-1	Aldrich	65 (*E*)	Active in GC-EAD[3,5]	HIV in cotton[4]., lilac flowers[5]	Present in an oviposition-deterrent blend[3]<br>Present in adult attractive blends[10,11]
**β-myrcene**	123-35-3	Fluka	95	Active in GC-EAD[3,5]	Cotton plants[8]	Present in an female attractive blend[10]
**3-carene**	13466-78-9	Aldrich	95			
**α-pinene**	80-56-8	Aldrich	98	1 ORN class[12]	Cotton plants[8]	
**β-pinene**	127-91-3	Fluka	99		Cotton plants[8]	
**(±)-linalool**	78-70-6	Aldrich	97	5 ORN classes[1,12], active in GC-EAD[3,5]	HIV in cotton13, lilac flowers[5]	Present in an oviposition-deterrent blend[3]
**geraniol**	106-24-1	¶	98	1 ORN class[2]		
**thymol**	89-83-8	Aldrich	99.5	1 ORN class[6]	Larval frass[6]	Present in an oviposition-deterrent blend[6]
**carvacrol**	499-75-2	Aldrich	98	2 ORN classes[12]	Larval frass[6]	Present in an oviposition-deterrent blend[6]
**α-humulene**	6753-98-6	Aldrich	98	2 ORN classes[1,12], active in GC-EAD[3]	Cotton plants[8]	
**β-caryophyllene**	87-44-5	Aldrich	98.5	2 ORN classes[12], active in GC-EAD[3]	Cotton plants[8]	
**α-copaene**	3856-25-5	Bedoukian	98	1 ORN class[12]		
**(*E*,*E*)-α-farnesene**	502-61-4	Bedoukian	99	1 ORN class[2], active in GC-EAD[3]	HIV in cotton[4].	Present in an oviposition-deterrent blend[3]
**(*E*,*E*)-farnesol**	106-28-5	Aldrich	95	1 ORN class[1]		
**(±)-nerolidol**	7212-44-4	Aldrich	98	2 ORN classes[1,12], active in GC-EAD[3]	Larval frass[6], damaged cotton plants[3]	Present in an oviposition-deterrent blend[6]
**(±)-phytol**	7541-49-3	Aldrich	99	Active in EAG[6]	Larval frass[6]	Present in an oviposition-deterrent blend[6]
**DMNT =** (3*E*)-4,8-dimethylnona-1,3,7-triene	19945-61-0	†	99	2 ORN classes[1], active in GC-EAD[3]	HIV in cotton[4]	Present in an oviposition-deterrent blend[3]<br>Deterrent to a female attractive blend[10]
**TMTT =** (3*E*,7*E*)-4,8,12-	62235-06-7	Aldrich	98	1 ORN class[1,2], active in GC-EAD[3]	HIV in cotton[4].	Present in an oviposition-deterrent blend[3]

trimethyltrideca-1,3,7,11-tetraene						
**sulcatone**	110-93-0	Aldrich	98	1 ORN class[2]		
**(*E*)-2-hexenal**	6728-26-3	Aldrich	98	6 ORN classes[1], active in GC-EAD[3]	Cotton plants[8]	
**nonanal**	124-19-6	Aldrich	95	Active in EAG[6] and in GC-EAD[5]	Larval frass[6]	Present in adult attractive blends[10,11]
**decanal**	112-31-2	Aldrich	99	2 ORN classes[12], active in GC-EAD[5]	Larval frass[6]	
**(*E*)-2-hexenol**	928-95-0	Aldrich	96	10 ORN classes[1]		Attractant for caterpillars[7]
**(*Z*)-3-hexenol**	928-96-1	Aldrich	98	8 ORN classes[1], active in GC-EAD[3]	Cotton plants[8]	
**1-hexanol**	111-27-3	Aldrich	98	8 ORN classes[1]		Attractant for caterpillars[7]
**1-heptanol**	111-70-6	Aldrich	99	5 ORN classes[1]		
**1-octanol**	111-87-5	Aldrich	99.5	3 ORN classes[1]		
**1-nonanol**	143-08-8	Aldrich	99.5	3 ORN classes[1]		
**(*Z*)-jasmone**	488-10-8	†	98	4 ORN classes[1], active in GC-EAD[3]	Cotton plants[8]	Attractant for caterpillars[7]
**methyl jasmonate**	39924-52-2	SAFC	98	Active in GC-EAD[3]	Damaged cotton plants[3]	
**(*Z*)-3-hexenyl acetate**	3681-71-8	Aldrich	98	5 ORN classes [1], active in GC-EAD[3,5]	HIV in cotton[4]	Present in an oviposition-deterrent blend[3]<br>Present in adult attractive blends[10,11]
**EDD** = ethyl (*E*,*Z*)-2,4-decadienoate	3025-30-7	Aldrich	98			
**14:OAc** = tetradecyl acetate	638-59-5	#		Active in EAG (males)[13]	Sex pheromone minor component[13,14]	
**(*Z*)-7-12:OAc** = (*Z*)-7-dodecen-1-yl acetate	14959-86-5	#		1 ORN class (females)[1]		
**(*Z*)-9-14:OAc** = (*Z*)-9-tetradecen-1-yl acetate	16725-53-4	#		Active in EAG (males)[13,15]	Sex pheromone minor component[13,14]	Reduces attraction at high doses[16]
**(*Z*)-9-14:OH** = (*Z*)-9-tetradecen-1-ol	35153-15-2	#		1 ORN class (males, females)[1,15]	Pheromone component of *S. exigua*[17]	Reduces attraction to the major component[16]
**(*Z*)-11-14:OAc** = (*Z*)-11-tetradecen-1-yl acetate	20711-10-8	#		Active in EAG (males)[13,15]	Sex pheromone minor component[13,14]	
**(*E*)-11-14:OAc** = (*E*)-11-tetradecen-1-yl acetate	33189-72-9	#		Active in EAG (males)[13,15]	Sex pheromone minor component[13,14]	
**(*Z*,*E*)-9,11-14:OAc** = (*Z*,*E*)-9,11-tetradecadien-1-yl acetate	50767-79-8	#		1 ORN class (males, females)[1,15]	Sex pheromone major component[13,14]	Necessary and sufficient for male attraction[16,18]
**(*Z*,*E*)-9,12-14:OAc** = (*Z*,*E*)-9,12-tetradecadien-1-yl acetate	30507-70-1	#		1 ORN class (males)[15]	Sex pheromone minor component[14]	Increases attraction at low doses[19]

¶ Gift from Prof. Monika Hilker, Berlin, Germany; † Gift from Prof. Wittko Francke, Hamburg, Germany; # Gift from Martine Lettere, Versailles, France

Supplementary References

1 Binyameen, M. *et al.* Spatial organization of antennal olfactory sensory neurons in the female *Spodoptera littoralis* moth: differences in sensitivity and temporal characteristics. *Chemical senses* **37**, 613-629, (2012).

2 Jonsson, M. & Anderson, P. Electrophysiological response to herbivore-induced host plant volatiles in the moth *Spodoptera littoralis*. *Physiological Entomology* **24**, 377-385, (1999).

3 Zakir, A. *et al.* Specific response to herbivore-induced de novo synthesized plant volatiles provides reliable information for host plant selection in a moth. *Journal of Experimental Biology* **216**, 3257-3263, (2013).

4 Paré, P. W. & Tumlinson, J. H. De Novo Biosynthesis of Volatiles Induced by Insect Herbivory in Cotton Plants. *Plant physiology* **114**, 1161-1167, (1997).

5 Saveer, A. M. *et al.* Floral to green: mating switches moth olfactory coding and preference. *Proceedings of the Royal Society of London B: Biological Sciences* **279**, 2314-2322, (2012).

6 Anderson, P. *et al.* Oviposition Deterring Components in Larval Frass of *Spodoptera Littoralis* (Boisd) (Lepidoptera, Noctuidae) - a Behavioral and Electrophysiological Evaluation. *Journal of Insect Physiology* **39**, 129-137, (1993).

7 Rharrabe, K., Jacquin-Joly, E. & Marion-Poll, F. Electrophysiological and behavioral responses of *Spodoptera littoralis* caterpillars to attractive and repellent plant volatiles. *Frontiers in Ecology and Evolution* **2**, (2014).

8 Loughrin, J. H., Manukian, A., Heath, R. R., Turlings, T. C. J. & Tumlinson, J. H. Diurnal cycle of emission of induced volatile terpenoids herbivore-injured cotton plants. *Proceedings of the National Academy of Sciences of the United States of America* **91**, 11836-11840, (1994).

9 Binyameen, M. *et al.* Identification of plant semiochemicals and characterization of new olfactory sensory neuron types in a polyphagous pest moth, *Spodoptera littoralis*. *Chemical senses* **39**, 719-733, (2014).

10 Saveer, A. M. *Recognition and modulation of olfactory signals in the noctuid moth Spodoptera littoralis*, Swedish University of Agricultural Sciences, (2012).

11 Borrero-Echeverry, F. *et al.* Flight attraction of *Spodoptera littoralis* (Lepidoptera, Noctuidae) to cotton headspace and synthetic volatile blends. *Frontiers in Ecology and Evolution* **3**, (2015).

12 Anderson, P., Hansson, B. S. & LÖFqvist, J. Plant-odour-specific receptor neurones on the antennae of female and male *Spodoptera littoralis*. *Physiological Entomology* **20**, 189-198, (1995).

13 Malo, E., Renou, M. & Guerrero, A. Analytical studies of *Spodoptera littoralis* sex pheromone components by electroantennography and coupled gas chromatography– …. *Talanta*, (2000).

14 Saveer, A. M. *et al.* Mate recognition and reproductive isolation in the sibling species *Spodoptera littoralis* and *Spodoptera litura*. *Frontiers in Ecology and Evolution* **2**, (2014).

15 Ljungberg, H., Anderson, P. & Hansson, B. S. Physiology and morphology of pheromone-specific sensilla on the antennae of male and female *Spodoptera littoralis* (Lepidoptera, Noctuidae). *Journal of Insect Physiology* **39**, 253 -260, (1993).

16 Campion, D. G. *et al.* Modification of the attractiveness of the primary pheromone component of the Egyptian cotton leafworm, *Spodoptera littoralis* (Boisduval) (Lepidoptera: Noctuidae), by secondary pheromone components and related chemicals. *Bulletin of entomological research* **70**, 417, (1980).

17 Acín, P., Rosell, G., Guerrero, A. & Quero, C. Sex pheromone of the Spanish population of the beet armyworm *Spodoptera exigua*. *Journal of chemical ecology* **36**, 778-786, (2010).

18 Quero, C., Lucas, P., Renou, M. & Guerrero, A. Behavioral responses of *Spodoptera littoralis* males to sex pheromone components and virgin females in wind tunnel. *Journal of Chemical Ecology* **22**, 1087-1102, (1996).

19 Dunkelblum, E., Kehat, M., Gothilf, S., Greenberg, S. & Sklarsz, B. Optimized mixture of sex pheromonal components for trapping of male *Spodoptera littoralis* in Israel. *Phytoparasitica* **10**, 21-26, (1982).
